# Back Pain in a Patient With Lung Cancer

**DOI:** 10.6004/jadpro.2012.3.3.10

**Published:** 2012-05-01

**Authors:** Lisa M. Doherty

**Affiliations:** From Dana-Farber Cancer Institute, Boston, Massachusetts

## History

Ms. P., a 62-year-old nonsmoker, was diagnosed with lung adenocarcinoma in April 2009. At the time of diagnosis, she underwent a left upper lobectomy for a moderately differentiated adenocarcinoma. Testing for epidermal growth factor receptor mutation revealed an exon 19 deletion, predicting a possible response for erlotinib (Tarceva). Following her lobectomy, Ms. P. received four cycles of cisplatin/vinorelbine chemotherapy and went on to receive radiation therapy to the mediastinum.

Ms. P. was well until March 2011, when she developed headaches and unsteadiness. MRI of the brain revealed a right cerebellar mass. She underwent resection in April 2011, and pathology results were consistent with metastatic adenocarcinoma. Postoperative MRI revealed improvement. Once she had recovered from surgery, Ms. P. enrolled in a clinical trial using cediranib concurrently with involved-field radiation therapy to the brain. This intervention was initiated in May 2011.

## Chief Complaint

Four months into her treatment with cediranib, Ms. P. developed pain in her lower back radiating down the left leg. She rated the pain 7/10 on most days. She had no complaints of bowel or bladder dysfunction, although she stated she did have a weak urine stream. Ms. P. added that she had bilateral tingling of the lower extremities with no apparent weakness. She also complained of a "bandlike" sensation radiating forward on both sides of the chest to the upper abdomen. Ms. P. reported that she had had several falls over the preceding 2 weeks, with no apparent injury.

## Review of Systems

On exam, Ms. P. was afebrile as well as alert and oriented to person, place, and time. She was inattentive at times and had notable short-term memory loss; language was intact. Her gait was steady but wide-based. She complained of tingling in her left lower extremity; no sensory abnormalities were appreciated. Strength was 5/5 in all extremities. Cardiac and respiratory exam results were all within normal limits. Ms. P. also had a spinal MRI, which can be seen in Figure 1.

## Choose the correct diagnosis:

- A: Bone metastasis
- B: Leptomeningeal metastasis
- C: Radiculopathy

## Scroll down for correct answer.

**Figure 1:**
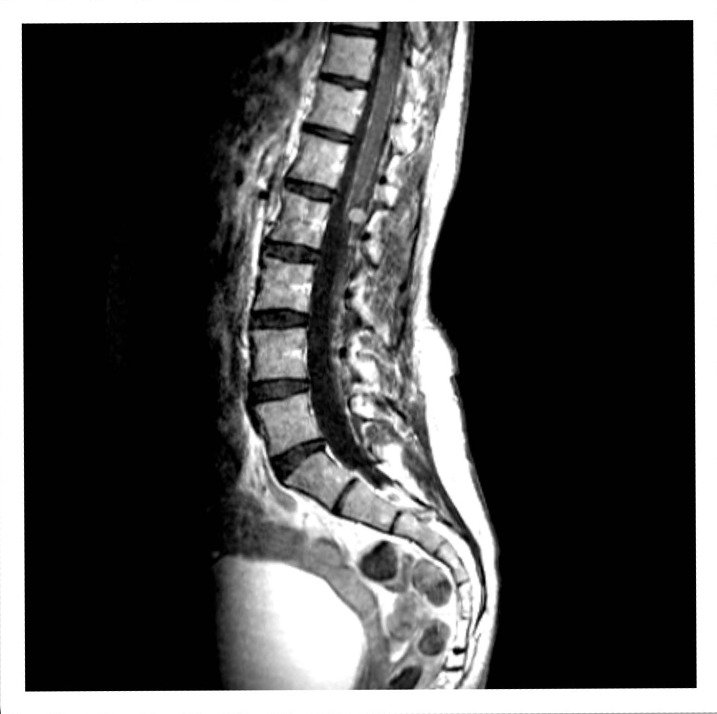


## Correct Answer

**Leptomeningeal metastasis** is diagnosed in 1% to 5% of patients with solid tumors, resulting in significant morbidity (Chamberlain, 2010). Leptomeningeal metastasis is more commonly seen in patients diagnosed with lung cancer, breast cancer, melanoma, cancers of the gastrointestinal tract, and cancers with unknown primary tumors who present with widespread systemic cancer, yet it can present after a disease-free interval (Clarke et al., 2010). Rarely is it the first manifestation of cancer in the absence of other systemic disease (Siddiqui, Marr, & Weissman, 2009). Ms. P. has leptomeningeal metastasis, as seen on the MRI of her spine (Figure 1). There is a nodular lesion at L2 as well as seeding along the cauda equina of the thoracic and lumbar regions of the spine.

## Explanation of Incorrect Answers

**Bone metastasis** occurs in about 30% to 40% of all non–small cell lung cancer patients (Coleman, 2001). Common complications from bone metastasis include bone pain, pathologic fractures, spinal cord compression, and malignant hypercalcemia. Impending spinal cord compression and vertebral fractures require urgent treatment with local-field external-beam radiotherapy. Percutaneous vertebroplasty should be considered to improve the patient’s quality of life (Rasulova et al., 2011). Ms. P.’s symptom of lower back pain could reflect possible bone metastasis, but given the lesion at L2 as well as seeding of the cauda equina (Figure 1) with no bony lesions present on the MRI of her spine, this is unlikely.

**Radiculopathy** is a condition in which the function of one or more of the nerve roots is affected (Tarulli & Raynor, 2007). The most common cause of radiculopathy is nerve root compression caused by either spondylosis or disc herniation, but it may also be caused by leptomeningeal metastasis. Pain that is not local and does not radiate is thought to arise from muscle, bone, or ligaments outside of the spinal canal (Groen, Baljet, & Drukker, 1990). Patients would typically report pain and sensory symptoms such as paresthesia, hyperesthesia, and dysesthesia that involve a specific dermatome (Chad, 2004). Ms. P. does present with signs of radiculopathy, such as the tingling of her lower extremities and a bandlike sensation of her abdomen, likely caused by her leptomeningeal metastasis, but this is not the primary diagnosis—merely a symptom of her disease. As mentioned above, Ms. P.’s symptoms are caused by leptomeningeal metastasis as seen on her spinal MRI (Figure 1).

## Management

The treatment of leptomeningeal metastasis is complicated by a shortage of standard treatments, poor sensitivity of diagnostic procedures, and a delay in diagnosis (Chamberlain, 2010). The median survival for patients with leptomeningeal metastasis is 4 to 6 weeks, although treatment can prolong this by preventing tumor growth for some time, improving neurologic symptoms and improving quality of life (Chamberlain, 2010).

Standard treatments include varying types of radiation to bulky disease when possible or whole-brain radiation if the patient has not yet had involved-field radiation to any bulky lesion. Systemic chemotherapy alone or in addition to intra–cerebrospinal fluid (CSF) chemotherapy may also be used and could potentiate improved quality of life with prolonged survival (Park et al., 2011).

## Follow-up

Ms. P. was treated with high-dose, once-weekly erlotinib at a dose of 1,200 mg weekly for 2 months starting in September 2011. Unfortunately, her tumor did not respond and the erlotinib was discontinued. A repeat lumbar puncture done in early December 2011 revealed the following abnormalities: elevated white blood cell count (14,000 cells/µL), red blood cells (200 cells/µL) in the CSF, low glucose (43), and elevated protein (81). Cytology was postive for atypical cells, thus suspicious for malignancy. She has since been on treatment with IV pemetrexed (Alimta) 500 mg/m^2^ every 3 weeks.
